# A Case of Systemic Lupus Erythematosus Presenting as Guillain-Barré Syndrome

**DOI:** 10.1155/2015/528026

**Published:** 2015-08-17

**Authors:** Helen Chioma Okoh, Sandeep Singh Lubana, Spencer Langevin, Susan Sanelli-Russo, Adriana Abrudescu

**Affiliations:** Icahn School of Medicine at Mount Sinai, Queens Hospital Center, Jamaica, NY 11432, USA

## Abstract

Systemic lupus erythematosus (SLE) is an autoimmune systemic disease with multiple organ involvement with high morbidity and mortality rate. Among the severe potential fatal complications are those of the central and peripheral nervous system which usually develop during the course of the disease and very rarely from the outset of the disease. We are reporting a rare case of Miller-Fisher (MFS) variant of Guillain-Barré syndrome (GBS) as the first manifestation of SLE in a 41-year-old female who progressed to flaccid paralysis with no neurological improvement with initial immunosuppressive therapy, plasmapheresis, and first cycle of intravenous immunoglobulin (IVIG) but with remarkable and complete recovery after the second 5-day course of IVIG.

## 1. Introduction

Neuropsychiatric systemic lupus erythematosus (NPSLE) as described by the American College of Rheumatology (ACR) research committee includes 19 neuropsychiatric syndromes divided into neurologic syndromes of the central, peripheral, and autonomic nervous system and the psychiatric syndromes observed in patients with SLE in which other causes have been excluded. These symptoms may precede the onset of SLE or can occur at any time during the course of SLE [1]. Peripheral nervous system involvement occurs in 3–18% [2]. Guillain-Barré syndrome which is classified under the peripheral involvement has been rarely associated with SLE. We report a case of Miller-Fisher variant of Guillain-Barré syndrome presenting as the initial presentation of SLE and while the nonneurological manifestations, renal function, and SLE serology resolved with immunosuppressive therapy and plasmapheresis, the MFS symptoms did not improve and the patient remained dependent on mechanical ventilation and endogastric tube feeding. Complete recovery was only achieved after the 2nd cycle of IVIG which was given approximately 2 months after the 1st cycle while only on prednisone maintenance daily dose.

## 2. Case Report

Patient is a 41-year-old female originally from Nigeria, immigrated to the United States 9 years ago with no past medical history, who presented to the emergency department with worsening lower extremity weakness and swelling for 3 months. She also complained of 2 days of right eye swelling with diplopia and blurry vision and 1-day history of an inability to walk. Patient reported a flu-like illness with diarrhea prior to symptoms 5 months ago. She works as a house remodeler and denied any exposure to mold, asbestos, heavy metals, and silica. She gave no history of skin rashes, mouth ulcers, hair loss, photosensitivity, or arthralgia. On initial physical examination vital signs were stable; oxygen saturation was 98% on room air. There was no evidence of skin rash, she denied joint tenderness, and there was no palpable swelling. Heart lungs and abdominal exam revealed no abnormalities. There was bilateral lower extremity pitting edema. The neurological exam revealed that she was alert and oriented x3, followed commands, and had no aphasia with intact comprehension and fluent speech. Cranial nerve examination revealed anisocoria right > left, extra ocular muscles intact, and no nystagmus and the rest of cranial nerves were normal. Motor exam revealed 2/5 strength in the proximal upper extremities (abduction and flexion) and 5/5 in wrist flexion and extension. Hip flexion was 2/5 on the right and 3/5 on the left. Knee extension and flexion, dorsiflexion, and plantar flexion were 5/5 bilaterally. Reflexes were equal and symmetric in upper extremities but diminished in lower extremities.

Laboratory data revealed leukopenia 4.3 K/mcl and anemia with Hg/Hct at 6.6/19.5 g/dL, respectively; platelets were within normal limit. Liver function panel was also within normal limits except albumin at 2.3 g/dL. Prealbumin was low at 8.1 mg/dL and BUN/creatinine was at 29/1.29 mg/dL, respectively. Urinalysis revealed protein of 300 and RBC of 11–25/hpf. Head CT, MRI brain, and C-spine and T-spine CT scans revealed no abnormalities. CT thorax was obtained which revealed moderate bibasilar pleural effusion and moderate pericardial effusion. Echocardiography revealed ejection fraction of 60%.

**Figure 1:**
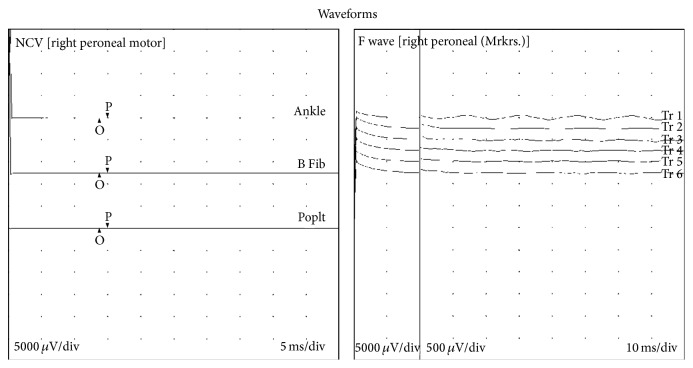
Nerve conduction studies (see Table 1). Findings: evaluation of the right peroneal motor nerve showed prolonged distal onset latency (13.8 ms), reduced amplitude (Ankle, 0.0 mV), reduced amplitude (B Fib., 0.0 mV), and reduced amplitude (Poplt., 0.0 mV). F wave studies indicate that the right peroneal F wave has no response.

Four days after admission, the patient's neurological status worsened. She became progressively weaker and lethargic and was only able to state her name. She had difficulty swallowing, speaking, taking deep breaths, and coughing. She was intubated for airway protection and placed on nasogastric tube (NGT) feeding. Neurologist evaluated the patient; exam was notable for absent reflexes in addition to progressive lower extremity weakness; GBS was suspected. She also developed ophthalmoparesis with inability of the eyes to cross the midline bilaterally. Stool studies were negative for* Campylobacter jejuni* and were guaiac negative. Lumbar puncture and plasma exchange were recommended. Cerebral spinal fluid (CSF) analysis revealed protein of 35 mg/dL; WBC was 8/cumm. Oligoclonal bands and myelin basic protein were absent, with Anti-GQ-1 antibody titers less than 1 : 100. West Nile viral titers were negative. Nerve conduction studies revealed absence of F wave response in right peroneal nerve, prolonged distal onset latency, and severe conduction block (Figure 1);[Table tab1] although CSF results were inconsistent with GBS as the protein was not elevated, a diagnosis was made clinically. Treatment with plasma exchange was begun 5 days after admission. She received 5 plasma exchanges with no improvement.

Due to the presence of leukopenia, pericardial and pleural effusions, proteinuria, and hematuria SLE work-up was sent with results showing the following: +ANA titers of 1 : 2560 4+ speckled, Anti-ds-DNA positive titer 1 : 80, and AntiSmith ab positive >8, low C3 and C4 levels at <40 mg/dL and <10 mg/dL, respectively. Serum antiribosomal P antibodies positive >8, serum antineuronal antibodies were negative, and lupus anticoagulant and anticardiolipin antibodies were negative. Patient fulfilled ACR SLE criteria and Pulse SoluMedrol therapy 1 gm daily for 3 consecutive days was started 7 days after admission. Renal function was rapidly deteriorating with anuria at 67 mL in 24 hours and worsening of BUN/creatinine to 50/3.29 mg/dL, respectively; hemodialysis (HD) was started 8 days after admission. Renal biopsy was performed which showed evidence of membranous and focal lupus nephritis [ISN/RPS 2004 classification lupus nephritis, classes III (A) and V], acute tubular necrosis (Figure 3).

SoluMedrol was given on alternate days with plasma exchange and hemodialysis to avoid removal and maintain optimal serum levels of steroids. SoluMedrol was continued as maintenance therapy on 120 mg IV daily.

Cyclophosphamide 500 mg every 2 weeks as per EURO Lupus protocol was started 10 days after admission due to lupus nephritis. Patient failed multiple weaning trials and subsequently needed PEG tube and tracheostomy tube. Plasma exchange was stopped after 5 sessions on day 14 of admission due to the absence of improvement in neurological status. Patient was started on IVIG on day 17 of admission. Patient received 1st course of IVIG 0.4 g/kg/day for 5 days concomitantly with SoluMedrol maintenance therapy on 120 mg IV daily and cyclophosphamide. Renal function began to improve with resolution of anuria; hemodialysis was discontinued. Due to significant SLE serology improvement with normalized C3 and C4 and negative ds DNA and improved renal function off hemodialysis, SoluMedrol was tapered to 40 mg IV daily.

**Figure 2:**
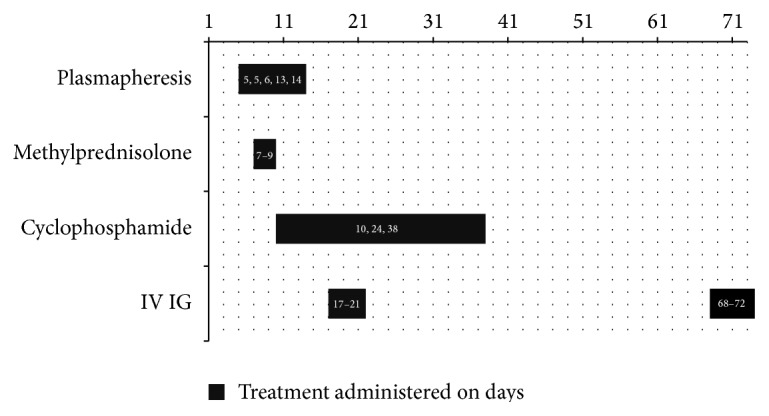


**Figure 3:**
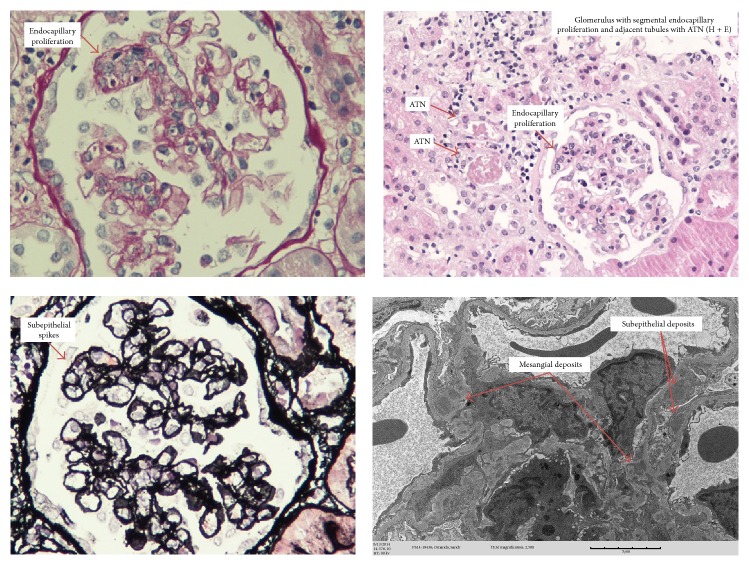
Kidney biopsy: membranous and focal lupus nephritis [ISN/PPS 2004 classification lupus nephritis, classes III (A) and V]; acute tubular necrosis.

Cyclophosphamide was terminated after the 3rd dose, 39 days after admission as the patient developed pancytopenia and fever with pneumonia and worsening of the sacral decubitus ulcer. In view of SLE with positive antiribosomal P protein and lack of improvement in the neurological/GBS symptoms (no improvement in muscle strength, ophthalmoparesis, or reflexes) and with patient still being dependent on mechanical ventilation and PEG tube feeding, a 2nd course of IVIG was started 2 g/kg divided over 5 days. Her weight was 60 kg and she was started on a 2nd course of IVIG 24 g/day, 68 days after admission. The choice of therapy and the onset of treatment are summarized in Figure 2.

Significant daily improvement in motor function and reflexes started to occur. Patient was able to start bedside physical therapy. Tracheostomy tube was subsequently removed as well as PEG tube. Patient was transferred to the inpatient acute rehabilitation unit for 2 weeks and was discharged home 131 days after initial presentation on prednisone 20 mg daily. Patient was seen in the rheumatology and neurology clinics within 1 month after discharge, with no significant residual motor or sensory deficits, walking without support, and asymptomatic for weakness. C3 and C4 levels were normalized at 100 mg/dL and 22.6 mg/dL, respectively. The patient was noted to have hematuria and low complement levels on subsequent clinic visits 2 months after and was started on mycophenolate mofetil in addition to prednisone.

## 3. Discussion

Guillain-Barré syndrome (GBS) is clinically defined as an acute peripheral neuropathy causing limb weakness that progresses over a time period of days or, at the most, up to 4 weeks. GBS is considered to be an autoimmune disease triggered by a preceding bacterial or viral infection.* Campylobacter jejuni*, cytomegalovirus, Epstein-Barr virus, and* Mycoplasma pneumoniae* are commonly identified antecedent pathogens [11]. The exact cause is unknown. Diagnosis of GBS includes clinical, serological, and electrophysiological criteria. The subtypes include acute inflammatory demyelinating polyneuropathy (AIDP) and acute motor axonal neuropathy (AMAN), acute motor and sensory axonal neuropathy (AMSAN), and Miller-Fisher variant with AIDP as the most common form of GBS in western countries and account for 85–90% of the patients with GBS. Miller-Fisher variant is characterized by a unique clinical triad of ophthalmoplegia, ataxia, and areflexia [12]. MFS variant is closely associated with antibodies against ganglioside GQ1b. The patient was diagnosed with SLE presenting as Miller-Fisher variant of Guillain-Barré despite Anti-GQ1b antibodies,* Campylobacter jejuni*, and CSF protein elevation being negative. The diagnosis of MFS still remains a clinical one [7]. Cranial nerve examination revealed anisocoria R > L, extra ocular muscles intact, and no nystagmus and the rest of cranial nerves were normal (Figure 1); although CSF results were inconsistent with GBS as the protein was not elevated, a diagnosis was made clinically and with the support of electroneuromyography.

Systemic lupus erythematosus (SLE) is an autoimmune disease of unknown etiology characterized by the presence of autoantibodies with involvement of multiple organ systems. Neuropsychiatric systemic lupus erythematosus (NPSLE) as described by the American College of Rheumatology (ACR) research committee includes 19 neuropsychiatric syndromes divided into neurologic syndromes of the central, peripheral, and autonomic nervous system and the psychiatric syndromes observed in patients with SLE in which other causes have been excluded These symptoms may precede the onset of SLE or can occur at any time during the course of SLE [1]. Peripheral nervous system involvement occurs in 3–18% [2].

Guillain-Barré syndrome as the initial presentation of SLE has been reported in only a few cases. [3, 4]. Some were treated with plasmapheresis and steroids with full recovery [3] or steroids, plasmapheresis, and cyclophosphamide with partial recovery [4] or had full recovery to only cyclophosphamide and steroids after failed initial treatment with IVIG and plasmapheresis [5] or had full recovery with cyclophosphamide and steroids after failed treatment with IVIG [6]. Reports from the literature document that some have had response to IVIG [7] or steroids only [8], 5 courses of plasmapheresis after failed treatment with IVIG, steroids, and cyclophosphamide [9], or 2 courses of plasmapheresis after failed treatment with IVIG and steroids [2]. Varying responses have been noted with each patient encountered and no universal treatment guidelines have yet been established. About 10% of GBS patients deteriorate after initial improvement or stabilization following IVIG or plasmapheresis treatment, a condition termed “treatment-related clinical fluctuation” [13]. However, no suggestions have been made regarding repeated treatment in this subgroup. A second course of IVIG was given as there was a case of severe unresponsive Guillain-Barré syndrome which responded to a repeat treatment of IVIG 2 g/kg given over 5 days, 14–21 days after presentation [10]. Therapeutic and outcome data have been summarized in Table 2.

An ongoing RCT (SID-GBS) to study the effects of a second course of IVIG in patients with poor prognosis is still ongoing in Netherlands [14]. A 2nd course of IVIG was given in this patient 51 days after the initial dose because of the absence of neurological improvement after the first course of IVIG and plasmapheresis despite the fact that, serologically, SLE seemed to be improving with normalization of C3 and C4 titers and Anti-ds-DNA. If a 2nd course of IVIG had been given sooner, perhaps an earlier response might have been elicited and possibly limiting hospital stay. Plasmapheresis and IVIG are both recommended treatments for GBS and both are considered equally effective. Corticosteroids alone do not alter the outcome of GBS [11]. We recommended from this case report when a diagnosis of SLE is made and is complicated by concurrent GBS with no neurological improvement after IVIG, plasmapheresis, or cyclophosphamide to consider a 2nd course of 5 days of IVIG with concomitant steroid use.

## Figures and Tables

**(a):** 

Site	NR	Onset	Norm. onset	O-P Amp.	Norm. O-P Amp.	Site 1	Site 2	Delta-0	Dist.	Vel.	Norm. Vel.
		(ms)	(ms)	(mV)				(ms)	(cm)	(m/s)	(m/s)
Right peroneal motor (Ext. Dig. Brev.)											
Ankle		**13.8**	<6	**0.0**	>3	B Fib.	Ankle	0.0	0.0		>40
B Fib.		13.8		**0.0**	>3	Poplt.	B Fib.	0.0	0.0		>40
Poplt.		13.8		**0.0**	>3						

**(b):** 

NR	F-Lat. (ms)	Lat. Norm. (ms)	L-R F-Lat. (ms)	L-R Lat. Norm.
Right peroneal (Mrkrs.) (EDB)				
**NR**		<60		<4

**Table 2:** 

Case report	Treatment	Outcome
Hsu et al. [3]	Plasma exchange and steroids	Ambulation 2 weeks after therapy

Chaudhuri et al. [4]	Plasma exchange, steroids, and cyclophosphamide	Ambulation with persistent foot drop bilaterally 4 months after admission

Santiago-Casas et al. [5]	Plasmapheresis + IVIG with no response. Cyclophosphamide + steroids with response after four weeks	Full clinical remission after four months of therapy

Van Larrhoven et al. [6]	IVIG with no response. Cyclophosphamide + high doses steroids with response	Resolution of symptoms began on day 45 to inpatient rehab on day 74. Symptoms resolved by day 90

Tan et al. [7]	IVIG	Follow-up at 2 months revealed a completely normal exam

Echaniz-Laguna et al. [8]	Steroids	Complete resolution of symptoms by day 40

Bingisser et al. [9]	IVIG, cyclophosphamide, and steroids with no response. Plasma exchange ×5 with response	After fifth plasma exchange patient's symptoms completely resolved

Hess et al. [2]	IVIG + steroids, with no response. Plasma exchange ×2 with response	Residual mild proximal weakness, otherwise neurologically normal

Farcas et al. [10]	IVIG. Retreatment with a second course of IVIG	Complete recovery
